# Universal access: making health systems work for women

**DOI:** 10.1186/1471-2458-12-S1-S4

**Published:** 2012-06-22

**Authors:** TK Sundari Ravindran

**Affiliations:** 1 Achutha Menon Centre for Health Science Studies, Sree Chitra Tirunal Institute for Medical Sciences and Technology, Medical College P.O, Trivandrum- 695 011, Kerala, India

## Abstract

Universal coverage by health services is one of the core obligations that any legitimate government should fulfil vis-à-vis its citizens. However, universal coverage may not in itself ensure universal access to health care. Among the many challenges to ensuring universal coverage as well as access to health care are structural inequalities by caste, race, ethnicity and gender. Based on a review of published literature and applying a gender-analysis framework, this paper highlights ways in which the policies aimed at promoting universal coverage may not benefit women to the same extent as men because of gender-based differentials and inequalities in societies. It also explores how ‘gender-blind’ organisation and delivery of health care services may deny universal access to women even when universal coverage has been nominally achieved. The paper then makes recommendations for addressing these.

## Background

**Figure 1:**
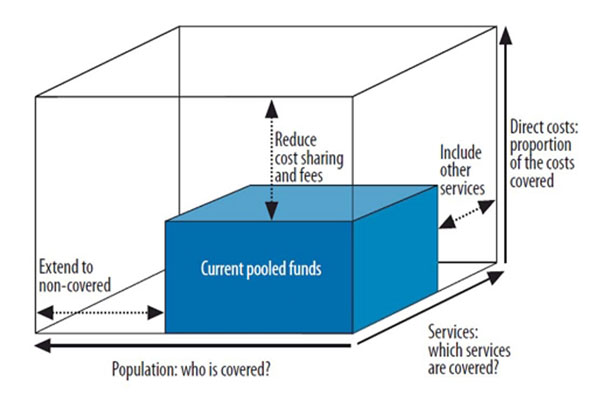
**Three dimensions to consider when moving towards universal coverage** Source: World Health Report 2010, p.12 [2]

Universal coverage is a term defined and understood differently by different people. In this paper, we use the term ‘universal coverage’ to mean that ‘*financing and organisational arrangements are sufficient to cover the entire population*, *removing ability to pay as a barrier to accessing health services and protecting people from financial risks *[1].’ ‘Coverage’ of a population essentially means that when the population seeks health care, they are assured of receiving at least a package of essential services at affordable or no cost at the point of service delivery. Achieving universal coverage calls for progress in three dimensions (fig 1). One is expanding the extent of financial protection available to the population, principally through reducing out-of-pocket expenditure. The second is putting in place organisational and financial mechanisms so that an increasing proportion of the population gets financial protection. The third is widening the range of services which are available at subsidised or no costs, so that services for which one will have to pay out-of-pocket (and hence face financial risks) are gradually minimised [2].

Universal access is a concept that includes but goes beyond universal coverage. It implies ‘*the absence of geographic*, *financial*, *organizational*, *socio-cultural and gender-based barriers to care *[1].’ In other words, universal access calls for making health systems functional and remove supply-side barriers, and for removing demand side barriers such as social exclusion and discrimination, lack of information and lack of decision-making power to seek health care.

Among the many challenges to ensuring universal coverage as well as access to health care are structural inequalities by caste, race, ethnicity and gender. This paper, based on a review of published literature, highlights ways in which policies aimed at promoting universal coverage may not benefit women to the same extent as men because of health system’s failure to recognise and address gender-based differentials and inequalities in societies. It also illustrates how, while universal coverage that factors in-gender may be a step in the right direction, universal access may still delude women because of gender-blind organisation and delivery of health care services. The paper then makes recommendations for addressing these.

## Methods

This paper draws on a larger publication prepared by this author for the World Health Organization on “Gender, Women and Primary Health Care Renewal” [3]. The search strategy adopted for information used in this paper consisted of searching Google, Medline and WHO websites using the following key words. For financial protection and population coverage, the key words used along side ‘gender’ and ‘women’ were as follows: health financing, health insurance, community-based health insurance, health micro-insurance, out-of-pocket health expenditure, catastrophic health expenditure, and health equity. For health care coverage, the key words used were gender or women and/or essential service-packages, priority-setting and health. In addition, literature pertaining to laws and policies on the availability in the public sector in health of contraceptive and abortion services was searched. For information pertaining to barriers to access in organisation and delivery of health services, the key words used included gender/women and/or health-seeking behaviour, service delivery, social exclusion, discrimination, provider-client (provider-patient) relationship, quality of care. The publications were scanned for information relevant to the analysis of universal coverage from a gender perspective, i.e. examined for their implications for women and men, boys and girls, of different social and economic groups and included publications that contained such information.

## Findings

### Gendered impact of Universal Coverage Reforms

This section queries universal coverage reforms from a gender lens and presents evidence that suggests that

• Women, especially those from vulnerable population groups, are more likely to be excluded in terms of population coverage

• Essential and routine health needs of women often do not find a place in the package of services covered

• As a consequence of both the above, women are more at risk of having unmet need for health care and/or less likely to receive adequate financial protection

### Coverage of women

In order to achieve universal coverage, health financing mechanisms in a country would have to reduce the proportion of out-of-pocket payment and increase the share of health expenditure financed by health insurance or other prepayment mechanisms [4]. Health insurance is a mechanism for risk pooling and cross-subsidising across income groups, and eliminating or substantially reducing out-of-pocket payment at the point of service delivery. Insurance and other prepayment schemes are therefore an important mechanism for financial protection. The universal coverage reform package in many countries consists of a combination of large scale compulsory health insurance – called Social Insurance – for those in formal employment; smaller scale voluntary micro-insurance schemes to cover those working in the informal sector and tax-revenue based financing to cover the poor and indigent [5,6].

*Social Insurance Schemes* are compulsory schemes consisting of payroll deductions of employees in the formal economic sector complemented by contributions by the employer and in some countries, by the government. The Schemes cover employees and at times, their dependents. A significant proportion of women globally do not participate in waged employment and those who do work mostly in the informal sector [7]. Social Insurance Schemes are therefore likely to exclude a vast majority of women except those who are covered as dependents of formal sector employees [8].

In many countries *Micro-insurance Schemes*, also known as Community-based Health Insurance (CBHI) mark the first stage in the transition to universal coverage, with the intention of consolidating the various funds after a certain stage. They are characterised by low premiums and low coverage that is typically provided to individuals that cannot afford/qualify for other insurance plans [9]. They are usually the route to cover those in the informal sector of the economy, and are also considered as an effective means of covering women for health care [10]. However, the poorest and those without access to cash –including the elderly and women from non-poor households- may be excluded from coverage even when the premium involved is small, both for reasons of non-affordability and of lack of information.

*Social Protection Health Schemes* are targeted interventions catering to vulnerable populations with a view to removing financial barriers to health care access. Fees and payments at the point of service delivery may be waived in specific health facilities, or vouchers and cash benefits may be provided to the targeted households. Social Protection Health Schemes are most common in Latin American and Caribbean countries, but are increasingly found in other parts of the world as well. A large number of these are focused on maternal and child health services. Studies carried out in countries where they have a long track record indicate that they are effective in removing financial barriers to health service access for low-income women [11-13]. While Social Protection Health Schemes expand coverage to poor women, women from non-poor households who may face financial constraints to seeking care still fall between the cracks.

*Conditional Cash-Transfers* are social protection schemes through demand-side financing. These typically consist of making cash payment to households or individuals from groups identified as under-served, conditional on their adopting desirable health behaviours, such as attendance for preventive health services by children and pregnant women. A review of six conditional cash transfer programmes in Latin America found that they consistently increased use of health services [14]. In the *Muthulakshmi Reddy Maternity Benefit Scheme* in Tamil Nadu, India, a substantial cash incentive is provided to low-income women conditional on institutional delivery, for the first two deliveries. The Scheme has increased rates of institutional delivery especially among the poorest women [15]. At the same time, women from the poorest and most socially marginalised households were disproportionately represented among those excluded from the Scheme. Reasons included pregnancy of higher than second order – thus not satisfying the eligibility criterion, but more importantly, of not being able to produce documentary proof of poverty status and of residence because of lack of information, time and resources [16].

Adolescent girls and older women are among subgroups of women often excluded from coverage. This is because of the almost exclusive focus on maternal health needs in services covered by prepayment schemes and Essential Service Packages (discussed in the next section).

### Coverage of health services essential for most women

“Essential Services Package” (ESP) consists of publicly funded services available at no cost or subsidised cost to those with ‘coverage’. An assessment of publicly financed Essential Services packages in about 152 countries during 1993-99, found that only 20 of 152 countries included within their ESPs all of the following: family planning, prenatal and delivery care, clean/safe delivery by trained attendants, post partum care and essential emergency obstetric care. Delivery care and emergency obstetric care were missing in a large number of Essential Services packages. Only 44 of the 152 included prevention of HIV/AIDS through condom promotion [17].

Safe abortion services are rarely part of the Essential Services Packages, although abortion is legal in almost all countries in specific circumstances, e.g. when the pregnancy is the result of rape or endangers the mother’s life. ESPs also rarely include services for reproductive health needs of men, older women and young women and men, and treatment for chronic diseases which affect a large proportion of women (and men).

Micro-insurance schemes are too small to achieve effective risk pooling and cross-subsidising to cover routine sexual and reproductive health services. They are “uninsurable” as stand-alone benefits because they are non-random and/or high probability events. Many micro-insurance schemes in South Asia and Latin America have been found to either not cover pregnancy; exclude coverage for the first pregnancy which has a higher risk of complications, or require a mandatory waiting period of 9 months before enrolling in the Scheme to prevent women from joining when they are pregnant [18].

### Financial protection

The extent of financial protection is linked to who is covered or excluded and for what services. In many countries, the uninsured have to pay out of pocket for health care services. Where there is a basic package of services financed by tax revenue and free at the point of service delivery, costs of other health services have to be met by out-of-pocket payment (OOP), or through a combination of different types of health insurance.

Women’s out-of-pocket payments has been found to be systematically higher than that of men [19] at least in part because of the high financial burden related to and paying for delivery care and other reproductive health services [20,21]. Another contributing factor may be the higher prevalence of a number of chronic diseases and mental health problems among women. High out-of-pocket expenses results in a higher proportion of women than men with unmet need for health services [22-24] not only among low income groups but in some settings, also among the wealthiest. For example, data from Latvia on unmet need for health services by sex and income quintile shows that while unmet need for health services was higher for the lowest income quintile and decreased with increasing income, women had a higher unmet need for health services than men in every income quintile [25]. Non-coverage of contraceptive services and other preventive or promotive sexual and reproductive health services is likely to contribute to unmet need for these services, judging from studies about the low willingness to pay for such services [26,27].

The extent of financial protection provided by Social Protection Health Schemes and Conditional Cash Transfers depends on the extent to which they cover associated non-medical expenses such as drugs and transportation. In the Safe Delivery Incentive Programme (SDIP) in Nepal payments, the cash incentive amount of NRP 1000 covered at most 25% of the cost of a normal delivery and only 5% of the cost of a caesarean section, not providing effective protection against catastrophic health expenditures [28].

### Achieving universal coverage of women: examples of Mexico and Thailand

#### Mexico

Mexico introduced the *Seguro Popular* or Popular Insurance in 2004 to expand coverage to nearly half the population who were not covered by the two insurance schemes in place for those in formal employment: the Mexican Social Security Institute (IMSS) and the Institute for Social Security and Services for State Workers (ISSSTE). Enrolment is voluntary. An annual fee is charged, ranging from US$ 60 to US$ 950 based on income levels, but the lowest income quintiles are exempted from payment [29].

The *Seguro Popular* provides access to 255 health interventions and drugs prescribed for these, covering 90% of medical conditions seen in outpatient departments. In addition, 18 high cost interventions are covered by a special fund. Interventions covered include a wide range of sexual and reproductive health services including mammography and cervical cancer screening as well as non-communicable diseases and mental health [29]. Entire families are enrolled in the Scheme ensuring that women are not excluded. Studies carried out within 2-3 years of implementation show an increase in deliveries by skilled birth attendants, mammography, cervical cancer screening and treatment of hypertension, among others [30] and an improvement in all indicators of financial protection [31].

#### Thailand

Thailand adopted Universal Health Care Coverage in 2001 to expand coverage to those who were not covered by existing insurance schemes. Financing is from tax revenue. Entire households are covered. The Benefits package is comprehensive and includes preventive, promotive, curative and rehabilitative services with a small number of exceptions. In 2006, ARV for HIV was included in the Benefits package and in 2008 renal replacement therapy was added on [32]. A wide range of sexual and reproductive health services are covered. However, safe abortion service is covered only for rape victims and for those whose health is at risk. Essential obstetric care is covered only for the first two deliveries.

Universal coverage successfully reduced incidence of catastrophic health expenditure from 5.4% in 2000 to 2% in 2006. Not a single household had experienced impoverishment due health expenditure in 21 of 76 provinces in 2008 [33]. A study covering 40,000 women in 2005-06 found that there were almost no rich-poor gaps in access to maternal health care and contraceptive services [34].

### Moving from universal coverage to universal access

Even when universal coverage has been achieved, universal access remains a challenge for many reasons including resource constraints, unequal distribution of existing health resources and organisational and managerial bottle-necks. Studies have also shown that in the absence of specific efforts to prioritise inclusion of those from marginalized sections of society, measures to achieve universal coverage could in fact widen inequities in health because access increases first in the better-off groups and then trickles down to the rest [35].

Gender-power inequalities which underlie women’s limited access to resources and lack of decision-making power are an important barrier to moving from universal coverage to universal access. For example, in six countries of the WHO-Western Pacific Region (Cambodia, Nauru, Philippines, Samoa, Solomon Islands and Tuvalu), 78- 97% of women aged 15-19 years had one or more problems in accessing health care when they were sick, many of these not related to affordability. About a fifth had problems in getting permission for treatment, 30-66% was not willing to go alone, and a majority in all countries except the Philippines were concerned that no female provider may be available [36]. A gender-sensitive health system can help women overcome many, though not all, of these barriers to health care access. This section presents information on health system-related barriers to access that women face.

#### Location and timing of services and modalities of delivery

Services available closer to home or workplace and at times of the day suitable to women are more likely to be utilised, and could make a big difference to identification of morbidity and effective treatment and cure. For example, studies from Bangladesh and Vietnam indicate the possibility of under-diagnosis and under-notification of women with tuberculosis, contributing to a lower-than-actual reported prevalence of tuberculosis in women [37-39]. A 2005 study carried out in a high TB incidence area in Peru found that when house-visits were made to the households of tuberculosis patients and their immediate neighbours, the odds of case-detection (as compared to self-reports) was 5.5 for those aged above 55 year and 3.9 for women [40].

Another example of the need for alternative strategies in order to be able to reach women is in the case of cataract-blindness. A much smaller proportion of women than men access cataract surgery, although the prevalence of cataract is found to be similar in men and women. In Southern China, an intervention providing free cataract testing in the community followed by low-cost and high quality surgery found that after five years of exposure to these interventions (2001-06), women were as familiar as men about cataract surgery and as willing to pay for surgery an amount of 500 Renminbi (RMB) or US$ 65. The gender differences found in the base-line had been reversed at the end of the five years [41].

Services that are much-needed and often unavailable at the primary care level are child-delivery services. The proportion of institutional deliveries in Tamil Nadu, India, increased from about 80% to 98% between 2004 and 2008. Almost all the increase in institutional deliveries was accounted for by deliveries in the newly operationalised 24x7 Primary Health Centres. The new users of institutional delivery services were from the poorest and most marginalised communities [15].

Simple changes in the organisation of services at the health facility level could also enhance access to care. In Nepal, the availability of DOTS services for tuberculosis in the same facility as maternal and child health care has been found to have increased case finding and successful treatment [42]. Integration of some services could enhance privacy and/or reduce stigma: as for example, when STI or HIV/AIDS services; abortion; or infertility services are made available in the sexual and reproductive health clinic.

#### Patient-provider interactions

For most patients, providers represent powerful authorities that are by training and social position far removed from their own lives and realities [43]. Patient perspectives on quality of care are shaped profoundly by the nature of their interaction with the health care provider.

Availability of a woman provider could make the difference between seeking and not seeking care for many women [37]. While this may be more widespread in traditional societies where segregation between the sexes is the norm, preference for a physician of the same sex is found also in other settings. A Netherlands study found that for physical examinations that required complete disrobing, were invasive or required examination of the genitalia, both women and men preferred to have a physician of the same sex [44].

There is a large literature on the physical and verbal abuse of women in labour and women seeking abortion and STI services by women health workers. A number of possible reasons for the abuse have been identified: the overworked health worker may be passing on her frustrations to patients; it may a case of discrimination against women from low-income or marginalised communities; and in the case of services such as contraception, abortion or STI/HIV care, the health worker may be judging the patient as having transgressed gender norms [43]. Immediate attention to preventing such abuse in the health care setting could make a big difference to women’s willingness to return for further care.

## Conclusions

Evidence examined in this paper, while not conclusive, indicates that universal coverage is likely to exclude more women than men, and even when achieved unlikely to translate into universal access to health care for women unless factors contributing to inequalities in affordability, availability and access to health care by women are systematically addressed. Based on the findings about the nature of barriers that women face, the following is a tentative agenda for action to make health systems work for women.

• When implementing insurance reforms, such as introduction of Social Health Insurance /Micro-Insurance, special attention is to be paid to coverage of women. Partially or fully subsidising premium payments for those who cannot afford to pay, and being aware that women, even when they belong to better-off households may fall within this category, would be important. Making households as the unit of enrolment in insurance schemes would help extend insurance coverage to women and other household members with low decision-making making power and financial resources.

• Social Protection Health Schemes and Conditional Cash Transfers should be free of conditionality and administrative procedures which may exclude the most marginalized women. Cash incentives made available as part of CCTs should be adequate to protect women from catastrophic health expenditures.

• For expanding ‘health care’ coverage to adequately cover women’s health needs, the range and content of services provided need to address differences between women and men in terms of conditions that occur exclusively in women or men; that are more common; manifest differently; more severe or with more serious consequences; and with different risk factors, for women or men.

• The range of reproductive health services in the Essential Services Package need to go beyond antenatal care and family planning, to include, at the least, skilled attendance at delivery and essential gynaecological services.

• Expanding health care coverage also calls for insurance schemes that are large enough to ensure effective risk pooling and cross-subsidising. Micro-insurance schemes will have to be subsidised to be able to include sexual and reproductive health services in the benefits package.

• Attention to gender differences in factors affecting health seeking behaviour should inform the location and timing of services. Services available closer to home or workplace and at times suitable to women or men are more likely to be utilised, and could make a big difference to identification of morbidity and effective treatment and cure.

• Changes towards more democratic and gender-sensitive provider-patient interactions are needed in the service -delivery setting. At the minimum, there should be no physical or verbal abuse of any patient by any member of the health team.

All of these are feasible given the political will. For, gender inequality is not a problem without a solution. What is needed is the political will and determination amidst policy makers at the highest level in international agencies and national governments [45].

## Competing interests

The author declares that she has no competing interests.
